# Untargeted metabolomics based on ultra-high performance liquid chromatography-mass spectrometry/MS reveals the lipid-lowering mechanism of taurine in hyperlipidemia mice

**DOI:** 10.3389/fnut.2024.1367589

**Published:** 2024-04-19

**Authors:** Xinzhe Guo, Tong Ou, Xinyu Yang, Qi Song, Lin Zhu, Shengquan Mi, Jing Zhang, Yanzhen Zhang, Wen Chen, Junxia Guo

**Affiliations:** ^1^Beijing Key Laboratory of Bioactive Substances and Functional Foods, Beijing Union University, Beijing, China; ^2^China National Center for Food Safety Risk Assessment, Beijing, China

**Keywords:** taurine, hyperlipidemia, lipid-lowering effect, metabolomics, ultra-high performance liquid chromatography-mass spectrometry (UPLC-MS)

## Abstract

**Introduction:**

Taurine has a prominent lipid-lowering effect on hyperlipidemia. However, a comprehensive analysis of the effects of taurine on endogenous metabolites in hyperlipidemia has not been documented. This study aimed to explore the impact of taurine on multiple metabolites associated with hyperlipidemia.

**Methods:**

The hyperlipidemic mouse model was induced by high-fat diet (HFD). Taurine was administered via oral gavage at doses of 700  mg/kg/day for 14  weeks. Evaluation of body weight, serum lipid levels, and histopathology of the liver and adipose tissue was performed to confirm the lipid-lowering effect of taurine. Ultra-high performance liquid chromatography-mass spectrometry (UPLC-MS)-based metabonomics analyses of serum, urine, feces, and liver, coupled with multivariate data analysis, were conducted to assess changes in the endogenous metabolites.

**Results and discussion:**

Biochemical and histological examinations demonstrated that taurine administration prevented weight gain and dyslipidemia, and alleviated lipid deposition in the liver and adipose tissue in hyperlipidemic mice. A total of 76 differential metabolites were identified by UPLC-MS-based metabolomics approach, mainly involving BAs, GPs, SMs, DGs, TGs, PUFAs and amino acids. Taurine was found to partially prevent HFDinduced abnormalities in the aforementioned metabolites. Using KEGG database and MetaboAnalyst software, it was determined that taurine effectively alleviates metabolic abnormalities caused by HFD, including fatty acid metabolism, sphingolipid metabolism, glycerophospholipid metabolism, diacylglycerol metabolism, amino acid metabolism, bile acid and taurine metabolism, taurine and hypotaurine metabolism. Moreover, DGs, GPs and SMs, and taurine itself may serve as active metabolites in facilitating various anti-hyperlipidemia signal pathways associated with taurine. This study provides new evidence for taurine to prevent hyperlipidemia.

## 1 Introduction

Hyperlipidemia, which is characterized by abnormal levels of lipids in blood, is recognized as one of the most important risk factors for cardiovascular diseases (CVD) and type 2 diabetes (T2D) (1, 2). Prevention and intervention of hyperlipidemia are crucial strategies to reduce the incidence of T2D and other chronic diseases. At present, pharmacological treatments such as statins and fibrates are commonly applied in the clinical treatment of hyperlipidemia, but these drugs often cause serious side effects such as musculoskeletal pain and sleep disturbance (3). Therefore, the development of new anti-hyperlipidemia active substances with fewer side effects is urgently needed.

Taurine (2-aminoethanesulfonic acid) is a conditionally essential amino acid that is abundantly present in mammalian plasma and various tissues such as heart, retina, liver, skeletal muscle, and platelets (4). Although taurine can be synthesized endogenously, the main source is still dietary intake, especially from seafood. Epidemiological studies across different countries have revealed that higher seafood consumption is associated with a reduced risk of developing metabolic diseases such as hyperlipidemia, hypertension, and diabetes (5, 6). Clinical studies indicates that the levels of plasma taurine are lower in subjects with obesity and diabetes (7). In contrast, taurine supplementation has been shown to increase plasma taurine and adiponectin levels, leading to significantly reduction in body weight, triglyceride (TG), total cholesterol (TC) and atherosclerotic index (AI) in obese patients (7, 8). Both animal (9) and human (10) studies have demonstrated that, with few exceptions, taurine generally does not produce any serious adverse effects even at high doses. At reasonable doses, taurine has an excellent safety profile and does not accumulate excessively in the body (11).

In addition to clinical studies, numerous animal experiments with rats, mice, hamsters, and rabbits have also confirmed the anti-hyperlipidemic effects of taurine (12–14). The mechanisms behind these effects are complex, with taurine exerting broad impacts on various molecular targets associated with lipid metabolism disorders (13). Taurine achieves this by inhibiting adipogenesis through the suppression of sterol regulatory element binding protein 1c (SREBP-1c), a major transcription factor for lipogenesis (15). Additionally, taurine increases energy expenditure by promoting peroxisome proliferator-activated receptor-gamma coactivator (PGC)-1α, the principal regulator of energy metabolism (16), and facilitates fatty acid β-oxidation by regulating adenosine monophosphate-activated protein kinase (AMPK), a key regulator of whole-body metabolism (17). Furthermore, taurine accelerates the biotransformation of cholesterol by promoting cholesterol 7a-hydroxylase (CYP7A1), the rate-limiting enzyme for bile acid synthesis (18). Taurine also enhances cholesterol excretion by regulating low density lipoprotein receptor (LDLR) and liver X receptor (LXR) (13). While the lipid-lowering mechanisms of taurine have been elucidated through the analysis of key signal molecules at the protein and gene levels, they do not fully account for taurine’s lipid-lowering effects. Metabolites, different from genes and proteins, reflect the environment of cells. In the cellular microenvironment, metabolites significantly influence cellular function through metabolite-macromolecule (enzyme and messenger RNA) interactions and pivotal covalent chemical modifications of DNA, RNA and proteins (19). Therefore, elucidating the influence of taurine on endogenous metabolites is crucial to understand taurine’s anti-hyperlipidemic effect.

Metabolomics is technology that assesses the dynamic changes of small molecules and metabolic pathways before and after stimulation. Recent mechanistic insights have shown all layers of the omics field are influenced by active metabolites, including the genome, epigenome, transcriptome and proteome (20, 21). Metabolomics has been widely used in pathophysiological pathway investigations, biomarker identification and active driver discovery (19, 22). In investigating the effect of taurine on hyperlipidemia-associated metabolites, only Kim and Bang (23). examined serum metabolites in dyslipidemic rats using an NMR-based metabolomics. Furthermore, the present study is a comprehensive analysis of the effects of taurine on metabolites in hyperlipidemic mice using ultra-high performance liquid chromatography-mass spectrometry (UPLC-MS). This technique offers superior chromatographic separation performance, high sensitivity and high resolution (24, 25).

In this study, a UPLC-MS-based metabolomics approach coupled with multivariate data analysis was used to employed to evaluate the changes in endogenous metabolites in serum, liver, urine and feces.

## 2 Materials and methods

### 2.1 Chemicals and assay kits

Taurine was provided by Qianjiang Yongan Pharmaceutical Co., Ltd. (Hubei, China). The TG kits (A110-1-1), TC kits (A111-1-1), LDL-C kits (A113-1-1), HDL-C kits (A112-1-1) and Oil Red O kits (D027-1-1) were purchased from Nanjing Jiancheng Bioengineering Institute (Jiangsu, China). Hematoxylin and eosin (H&E) were purchased from Beijing Dingguochangsheng Biotechnology Co., Ltd. (Beijing, China). Methanol, acetonitrile, and formic acid (all MS grade) were purchased from Fisher Scientific (Fisher Scientific, Pittsburgh, PA, United States). The ultra-pure water was purified by a Milli-Q ultrapure water system (Merck Millipore, Milford, MA, United States). CNWBOND HC-C18 solid phase extraction (SPE) cartridge was purchased from Anpel Laboratory Technologies Inc. (Shanghai, China).

### 2.2 Animals and dosage information

Male C57BL/6J mice (8 weeks old) were purchased from Beijing HFK Bioscience Co. Ltd. (HFK, Beijing, China). All animals were housed at a temperature of 22 ± 2°C, relative humidity of 50% ± 10% with food and water *ad libitum*, and a 12 h light-dark cycle was maintained. Two or three animals were housed in each cage to ensure social behavior and equal access to food. After one-week acclimation, all mice were randomly divided into three groups of 9 mice each: a control group (CON), a model group (MOD), a taurine group (TAU). The TAU group was given 700 mg/kg/day of taurine by intragastrical administration for 14 weeks, and the other groups were given the same amount of distilled water. The CON group was fed with a standard diet (10% kcal from fat, 70% kcal from carbohydrate, 20% kcal from protein; 1,022; HFK, Beijing, China); the MOD group and TAU group were fed with a high-fat diet (41% kcal from fat, 43% kcal from carbohydrate, 16% kcal from protein; H10141; HFK, Beijing, China).

This research protocol was approved by the Experimental Animal Ethics Committee of the Functional Test Center for Health Food, College of Arts and Sciences, Beijing Union University (20220301). Animal welfare and experimental procedures were strictly performed in accordance with the Guidelines for the Care and Use of Laboratory Animals.

### 2.3 Experimental design

Food intake and body weight were recorded daily during the experimental period. Before the end of week 14, each mouse was transferred to a metabolic cage (1 mouse/cage), 24 h feces samples and 12 h urine samples were collected from individual metabolic cages. The urine samples were centrifuged at 3,000 rpm for 10 min at 4°C, and the supernatants were collected. Both feces and urine supernatants were stored at −80°C prior to analysis. At study termination, mice were sacrificed in a 10 h fasted state under isoflurane anesthesia. The blood samples were standing at room temperature for 1 h and then centrifuged at 3,000 rpm for 10 min to obtain serum, which was stored at −80°C. And a segment of the liver was immediately frozen and stored at −80°C, while the remainder of the liver and epididymal adipose tissues were fixed in a 4% paraformaldehyde solution for 48 h.

### 2.4 Serum biochemical assays and histopathological assessment

The serum levels of TC, TG, low-density lipoprotein cholesterol (LDL-C), and high-density lipoprotein-cholesterol (HDL-C) were tested with biochemistry assay kits according to the manufacturer’s instructions.

The fixed liver and adipose tissue samples were embedded in paraffin sectioned into 4 μm slices, and stained with hematoxylin and eosin (H&E). After deparaffinization and rehydration, the sections were stained with Harris hematoxylin solution and differentiated using 1% acid alcohol. Subsequently, the sections were stained with eosin solution for 3 min, dehydrated with graded alcohol, and cleared in xylene. Finally, the sections were scanned using a slide scanner.

In the Oil-Red O staining experiment, the liver tissues were embedded in the OCT medium and then cut into 7 μm sections. Subsequently, the frozen liver sections were immersed in 10% formaldehyde solution for 10 min and washed three times with distilled water. The sections were then stained in 0.5% Oil-Red O in 60% isopropanol for 5 min, counterstained by hematoxylin, washed, sealed in neutral gum, and observed under an optical microscope.

### 2.5 Metabolomics sample preparation

Twenty microliters of serum samples were added to the activated CNWBOND HC-C18 SPE cartridge and eluted with 3 mL of methanol, and the eluate was collected. Subsequently, the serum samples were vortexed and centrifuged at 15,000 rpm for 20 min and the supernatant was collected for UPLC-MS analysis.

For urine samples, 1 mL supernatant was filtered through a 0.22 μm aqueous filter after thawing on ice. The filtered urine samples were then centrifuged at 15,000 rpm for 20 min and the supernatant was collected for UPLC-MS analysis.

Fecal samples (0.5 g) were lyophilized, pulverized, and mixed with 30 mL methanol (4°C), followed by sonication for 30 min. The mixture was then vortexed and centrifuged at 12,000 rpm for 15 min. The supernatant was collected and filtered through a 0.22 μm organic filter. Subsequently, the filtered supernatant was rotated in a rotary evaporator for 1 h and reconstituted with methanol. The redissolved liquid was then centrifuged at 15,000 rpm for 20 min, and the supernatant was used for UPLC-MS analysis.

For liver tissues, 0.5 g of the tissues were homogenized in 3 mL of cold methanol, further ultrasonically broken for 30 min, and then centrifuged at 12,000 rpm for 15 min. After using activated CNWBOND HC-C18 SPE cartridges, samples were collected and centrifuged at 15,000 rpm for 20 min, and the supernatant was used for UPLC-MS analysis.

As a part of the system conditioning and quality control process, a pooled quality control sample (QC) was prepared by mixing equal volumes of blood, urine, feces and liver tissue samples. The QC samples were disposed and tested in the same manner as the analytic samples.

### 2.6 UPLC-MS conditions

The UPLC separation was conducted using a Thermo Vanquish Flex Binary RSLC platform (Thermo Fisher Scientific, Waltham, MA, United States) equipped with a diode array detector (DAD). Chromatographic separation was performed on a Thermo Accucore a Q C18 (150 × 2.1 mm, 2.6 μm; Thermo Fisher Scientific, Waltham, MA, United States). The column temperature was maintained at 40°C, while the autosampler temperature was set at 4°C. The 0.1% formic acid aqueous solution (v/v, A) and methanol (B) were regarded as the mobile phase with a flow rate of 0.3 mL/min. The gradient elution was performed as follows: 1–45% B at 0–1 min; 45–56% B at 1–5 min; 56–70% B at 5–11 min; 70–75% B at 11–12 min; 75–100% B at 12–22 min, 100% B at 22–32 min, 100–0% B at 32–33 min. The injection volume was set at 2 μL. In addition, one QC sample was injected per five samples to supervise the system stability throughout the UPLC-MS analysis.

The UPLC-Orbitrap-MS/MS detection was conducted on a Q Exactive Plus mass spectrometer (Thermo Fisher Scientific, Waltham, MA, United States). Mass spectrometry was performed in electrospray ionization in positive (ESI^+^) and negative (ESI^−^) ion modes with a full-scan mass range of 100–1,500 m/z. The heated electrospray ionization sources were set as follows: spray voltages of 4.0 kV in ESI^+^ and 3.5 kV in ESI^−^; source heater temperature, 320°C; capillary temperature, 350°C/380°C (positive/negative); sheath gas flow, 50 arb; auxiliary gas flow, 10 arb; and S-lens RF level, 55. The UPLC-MS/MS data were analyzed using Xcalibur 4.1 software (Thermo Fisher Scientific, Waltham, MA, United States).

### 2.7 Multivariate statistical analysis and identification of potential differential metabolites

The UPLC-MS raw data were imported into Progenesis QI software (Nonlinear Dynamics, Waters, United States) software for peak detection, noise filtering and automatic alignment. Subsequently, retention times (RT), mass-to-charge ratio (m/z) values and normalized peak area were derived, and a data matrix constructed. The mass spectra of these metabolic features were identified by using the accurate mass, MS/MS fragment spectra, and isotope ratio differences through searching in reliable biochemical databases such as the Human metabolome database (HMDB)^1^ and the Metlin database.^2^ The standardized UPLC-MS data matrix underwent multivariate statistical analysis, including principal component analysis (PCA) and orthogonal partial least squares discriminant analysis (OPLS-DA). The PCA method was used to determine the differences among the CON group, the MOD group and the TAU group. The OPLS-DA method was validated by 200 permutation tests. The significantly differential metabolites were further screened based on the variable importance projection (VIP) obtained from the OPLS-DA model and the *p*-value of the student’s *t*-test. Metabolites with VIP >1.5 and *p* < 0.05 were taken as potential differential metabolites.

### 2.8 Pathway analysis

In order to explore the metabolic effects of taurine on hyperlipidemia, differential metabolites were demonstrated using metabolic enrichment and pathway analysis by the Python package Scipy stats^3^ and the KEGG database.^4^ These differential metabolites were then input into MetaboAnalyst 5.0 software^5^ to construct the most affected metabolic pathways.

### 2.9 Statistical analysis

The results of body weight and serum lipids were expressed as means ± standard deviation (SD). Statistical differences among groups were calculated by unpaired two-tailed student’s *t*-tests. *p* < 0.05 was considered statistically significant.

## 3 Results

### 3.1 Taurine improved serum lipid profile and tissue lipid deposition in hyperlipidemia mice

**Figure 1:**
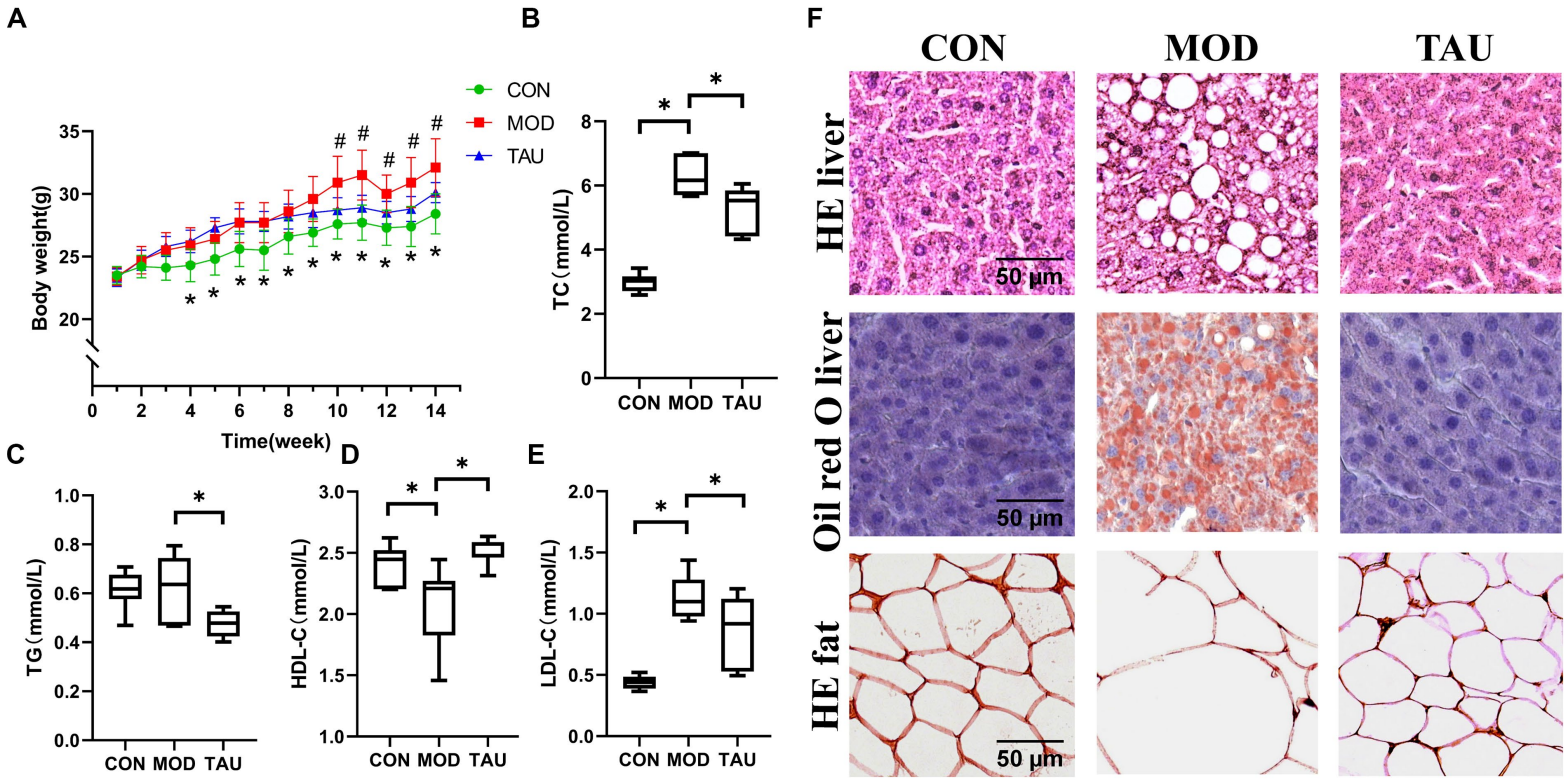
Taurine prevented dyslipidemia and alleviates lipid accumulation of organs in hyper-lipidemic mice. **(A)** Body weight; **(B)** serum total cholesterol (TC); **(C)** serum triglyceride (TG); **(D)** serum high-density lipoprotein cholesterol (HDL-C); **(E)** serum low-density lipoprotein cholesterol (LDL-C); and **(F)** histopathology. ^*^p < 0.05 compared to CON group, ^#^*p* < 0.05 compared to MOD group.

There was no significant effect of taurine administration on food intake throughout the treatment period (Supplementary Figure S1). As shown in Figures 1A–E, high-fat diet significantly increased the body weight and serum TC, TG and LDL-C levels of mice, while obviously reducing the level of HDL-C of mice in MOD group, indicating that the hyperlipidemia model has been successfully established. While, taurine treatment significantly prevented all these changes caused by hyperlipidemia.

To confirm the effect of taurine on tissue morphology and fat deposition, adipose and liver tissue sections were stained by H&E and/or Oil Red O (Figure 1F). The no histological abnormalities were found in the liver sections of the CON group. While, in the MOD group, a large amount of fat accumulation and vacuolization in the hepatocytes were seen in the liver stained with H&E, along with numerous lipid droplets observed in the hepatocytes stained with Oil Red O. In the TAU group, compared to the MOD group, improvements in cell morphology of the liver and adipose tissue were noted, with a significant reduction in liver vacuoles and lipid droplets.

The above results show that taurine has an excellent preventive effect on the changes of blood lipid profile and tissue lipid accumulation in hyperlipidemic mice induced by high-fat diet.

### 3.2 Metabolomic analysis

#### 3.2.1 Analysis and screening of differential metabolites in serum samples by UPLC-MS based metabolomics technology

**Figure 2:**
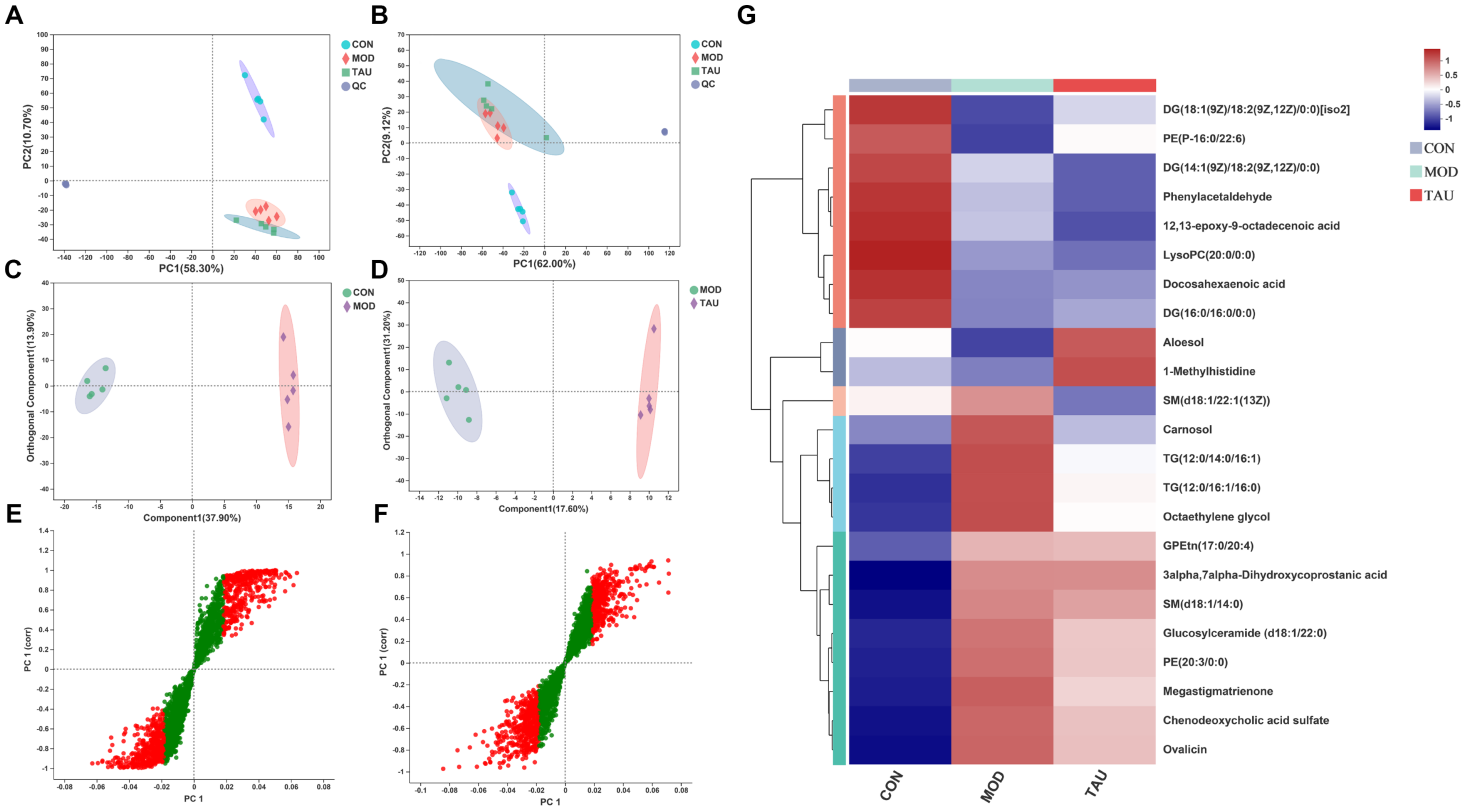
Multivariate statistical analysis of UPLC-MC-based effects for taurine on serum from high-fat-fed mice and heatmaps of the identified metabolites. Scatter plot of PCA scores in positive **(A)** and negative **(B)** ion mode; **(C)** OPLS-DA score scatter plot comparing CON and MOD; **(D)** OPLS-DA score scatter plot comparing TAU and MOD; **(E)** S-plots comparing CON and MOD; **(F)** S-plots comparing TAU and MOD. **(G)** Hierarchical clustering heat map of the differential metabolites between CON, MOD, and TAU groups.

With the application of UPLC-MS analysis, the representative total ion chromatograms (TIC) of serum samples from all three groups in ESI^+^ and ESI^−^ ion modes were presented in Supplementary Figure S2. The overlapped TIC from the QC samples in ESI^+^ and ESI^−^ ion modes were presented in Supplementary Figure S3. The results confirmed the reliability and repeatability of this method in large batches of samples. The PCA method was used to evaluate the repeatability of serum samples data and metabolomic differences in samples of 3 groups (Figures 2A,B). The established PCA model provided excellent prediction and interpretation ability. In the PCA scatter plot, QC samples were highly clustered, reflecting the stability and repeatability of the detection system. Because QC samples were prepared from a mixture of serum, liver, feces, and urine samples while PCA was performed separately for each single type of tissue sample, QC samples were clustered in non-central locations on the PCA score plot. The MOD group and the CON group were separated in the PCA score scatter plot of serum samples (Figures 2A,B), indicating that the mouse hyperlipidemia model was successfully established. Moreover, a clear separation of the TAU group and MOD group was found in the positive and negative modes (Figures 2A,B).

To explore the metabolites responsible for the separation between groups, we constructed OPLS-DA models between CON, MOD, and TAU groups in pairs. As shown in Figures 2C,D, established models exhibited excellent interpretability and prediction (CON vs. MOD, positive-ion, R2Y = 0.996, Q2 = 0.947; TAU vs. MOD, R2Y = 0.995, Q2 = 0.732), and the data was not overfitted. The CON group and the MOD group were significantly separated in the OPLS-DA score chart, which indicated that there were significant metabolite differences between the two groups. To reveal the potential differential metabolites that contribute to differences between groups, we generated S-plots following the OPLS-DA model (Figures 2E,F). The metabolites with the VIP >1.5 based on the above OPLS-DA models and *p* < 0.05 were selected as potential differential metabolites preliminarily. According to the differential metabolites identification method above section 2.7, 23 potential differential metabolites were identified in serum samples, which were listed in Table 1 with corresponding m/z, VIP, retention time, and associated trends. Compared with the CON group, the HFD resulted in an increase of 13 differential metabolites and a decrease of 9 differential metabolites. While taurine treatment partially prevented the above abnormal changes of metabolites caused by the HFD. Figure 2G presents a heat-map and cluster analysis of significantly varying metabolites in different groups.

**Table 1:** Trend of significantly differential metabolites in serum.

No.	Sort	Metabolite	Formula	Mode	Adducts	RT	m/z	VIP	Trend	
									MOD/CON	TAU/MOD
1	Triglyceride	TG (12:0/16:1/16:0)	C_47_H_88_O_6_	Pos	M + NH_4_	32.02	771.6470	2.14	↑^***^	↓^**^
2	Triglyceride	TG (12:0/14:0/16:1)	C_45_H_84_O_6_	Pos	M + NH_4_	30.06	743.6161	2.13	↑^***^	↓^**^
3	Triglyceride	TG (16:0/16:0/18:2)	C_53_H_98_O_6_	Pos	M + Na	29.47	853.7251	1.69	—	↓^*^
4	Sphingolipids	SM [d18:1/22:1 (13Z)]	C_45_H_89_N_2_O_6_P	Neg	M + FA − H	27.13	829.6445	1.56	↑^**^	—
5	Sphingolipids	SM (d18:1/14:0)	C_37_H_75_N_2_O_6_P	Neg	M + FA − H	23.93	719.5368	2.15	↑^***^	↓
6	Sphingolipids	Glucosylceramide (d18:1/22:0)	C_46_H_89_NO_8_	Neg	M − H	26.07	828.6573	2.12	↑^***^	↑
7	Glycerophospholipids	PE (P-16:0/22:6)	C_43_H_74_NO_7_P	Pos	M + H	24.37	748.5271	1.51	↓^***^	↑^**^
8	Glycerophospholipids	PE (20:3/0:0)	C_25_H_46_NO_7_P	Neg	M − H	17.51	502.2983	2.15	↑^***^	↓
9	Glycerophospholipids	LysoPC (20:0/0:0)	C_28_H_58_NO_7_P	Neg	M + FA − H	21.43	596.3935	1.57	↓^***^	↑
10	Glycerophospholipids	GPEtn (17:0/20:4)	C_42_H_76_NO_8_P	Neg	M − H	24.56	752.5226	1.67	↑^**^	↓
11	Fatty acid	Docosahexaenoic acid	C_22_H_32_O_2_	Pos	M + H	18.78	351.2294	1.62	↓^***^	↑^**^
12	Fatty acid	3alpha,7alpha-dihydroxycoprostanic acid	C_27_H_46_O_4_	Neg	M − H	20.53	433.3323	2.81	↑^***^	↓^***^
13	Fatty acid	12,13-epoxy-9-octadecenoic acid	C_18_H_32_O_3_	Pos	M + H	15.86	297.2422	1.52	↓^***^	↑^*^
14	Diglycerides	DG [18:1 (9Z)/18:2 (9Z, 12Z)/0:0](iso2)	C_39_H_70_O_5_	Pos	M + H	25.31	551.5032	2.93	↓^***^	↑^*^
15	Diglycerides	DG (16:0/16:0/0:0)	C_35_H_68_O_5_	Pos	M + H − H_2_O	25.91	551.5032	1.57	↓^**^	—
16	Diglycerides	DG [14:1 (9Z)/18:2 (9Z, 12Z)/0:0]	C_35_H_62_O_5_	Pos	M + NH_4_	23.8	585.4492	1.88	↓^**^	—
17	Bile acid	Chenodeoxycholic acid sulfate	C_24_H_40_O_7_S	Pos	M + H	3.36	490.2864	2.43	↑^***^	↓^*^
18	Amino acids	1-Methylhistidine	C_7_H_11_N_3_O_2_	Pos	M + H	0.97	170.0928	1.59	↓	↑^*^
19	Organooxygen compounds	Octaethylene glycol	C_16_H_34_O_9_	Pos	M + H	3.18	388.2545	1.88	↑^***^	↓^**^
20	Organooxygen compounds	Megastigmatrienone	C_13_H_18_O	Pos	M + H	9.79	191.1433	1.63	↑^***^	↓^*^
21	Other	Phenylacetaldehyde	C_8_H_8_O	Pos	M + H	10.46	121.0651	1.79	↓^**^	↓^*^
22	Other	Ovalicin	C_16_H_24_O_5_	Pos	M + H − 2H_2_O	5.78	319.1521	2.55	↑^***^	↓^*^
23	Other	Carnosol	C_20_H_26_O_4_	Pos	M + NH_4_	11.62	353.1728	1.97	↑^**^	↓^*^

^*^p < 0.05, ^**^p < 0.01, and ^***^p < 0.001. CON, the control group; MOD, the model group; TAU, the taurine group.

#### 3.2.2 Screening and multivariate analysis of differential metabolites in liver, feces and urine samples

Metabolomic data from liver, urine, and fecal samples were processed and analyzed as described in section 3.2.1. The results demonstrated taurine’s preventive effect on the hyperlipidemic mice, which was similar to those of the serum samples. Typical TIC plots of liver, fecal, and urine samples in the positive and negative models were presented in Supplementary Figures S4–S6. The relevant PCA scores scatter plots, OPLS-DA scores scatter plots, and S plots of liver, urine, and feces samples were shown in Supplementary Figures S7–S9, respectively, and the evaluation parameters of all models met the requirements. Subsequently, 26 potential differential metabolites in the liver, 6 in urine, and 21 in feces were identified according to the above criteria and treatment methods (Tables 2–4). Compared with the CON group, the HFD resulted in an increase in 21, 10, and 2 differential metabolites and a decrease in 2, 11, and 3 differential metabolites in the liver, feces and urine, respectively. While taurine treatment partially prevented the above abnormal changes of differential metabolites caused by HFD. Hierarchical clustering heat maps of these differential metabolites of these metabolites were shown in Supplementary Figures S7–S9.

**Table 2:** Trend of significantly differential metabolites in liver.

No.	Sort	Metabolite	Formula	Mode	Adducts	RT	m/z	VIP	Trend	
									MOD/CON	TAU/MOD
1	Glycerophospholipids	PE (22:6/P-18:0)	C_45_H_78_NO_7_P	Neg	M − H	25.26	774.5426	3.13	↑^***^	↓^*^
2	Glycerophospholipids	GPCho (16:1/16:1)	C_40_H_76_NO_8_P	Pos	M + Na	24.3	752.5204	3.29	↑^***^	↓^*^
3	Glycerophospholipids	PC (14:0/0:0)	C_22_H_46_NO_7_P	Pos	M + H	15.86	468.3087	2.45	↑^***^	↓^*^
4	Glycerophospholipids	LysoPE (16:1 (9Z)/0:0)	C_21_H_42_NO_7_P	Pos	M + H	16.11	452.2773	3.39	↑^***^	↓
5	Glycerophospholipids	GPEtn (18:0/18:1)	C_41_H_80_NO_8_P	Neg	M − H	25.86	744.5552	2.31	↑^***^	↓
6	Glycerophospholipids	GPEtn (16:1/20:4)	C_41_H_72_NO_8_P	Neg	M − H	23.5	736.4930	2.44	↑^***^	↓
7	Glycerophospholipids	CL (a-13:0/a-25:0/18:2/18:2)	C_83_H_15_4O_17_P_2_	Pos	M + H + Na	25.1	754.5362	3.88	↑^***^	↓^*^
8	Glycerophospholipids	PC (22:4/0:0)	C_30_H_54_NO_7_P	Pos	M + H	18.51	572.3713	2.42	—	↓^**^
9	Glycerophospholipids	PS [16:0/18:2 (9Z, 12Z)]	C_40_H_74_NO_10_P	Neg	M + Na − 2H	23.61	780.4806	1.68	↑^*^	↓
10	Glycerophospholipids	PI [16:0/22:5 (7Z, 10Z, 13Z, 16Z, 19Z)]	C_47_H_81_O_13_P	Pos	M + ACN + Na	23.7	948.5567	2.73	↑^*^	↓^*^
11	Glycerophospholipids	PE (16:0/0:0)	C_21_H_44_NO_7_P	Pos	M + H	17.71	454.2928	1.87	↑^**^	↓^*^
12	Glycerophospholipids	GPCho (17:1/20:5)	C_45_H_78_NO_8_P	Pos	M + Na	24.44	814.5352	2.88	↑^**^	↓
13	Glycerophospholipids	Choline Glycerophosphate	C_8_H_20_NO_6_P	Pos	M + H	1.00	258.1105	1.89	↓^*^	—
14	Diglycerides	DG [16:1 (9Z)/16:1 (9Z)/0:0]	C_35_H_64_O_5_	Pos	M + H	24.37	587.4643	1.92	↑^***^	↓^*^
15	Diglycerides	DG [16:0/16:1 (9Z)/0:0](iso2)	C_35_H_66_O_5_	Pos	M + H	25.07	589.4800	2.94	↑^***^	↓^*^
16	Diglycerides	DG [15:0/20:5 (5Z, 8Z, 11Z, 14Z, 17Z)/0:0]	C_38_H_64_O_5_	Pos	M + CH_3_OH + H	23.24	633.5068	2.42	↑^***^	↓^**^
17	Diglycerides	DG [12:0/18:1 (9Z)/0:0](iso2)	C_33_H_62_O_5_	Pos	M + H	24.26	561.4488	3.39	↑^***^	↓^*^
18	Amino acids	Tiglylglycine	C_7_H_11_NO_3_	Neg	M − H	3.00	156.0667	2.89	—	↑^*^
19	Amino acids	2-Methylindole	C_9_H_9_N	Pos	M + H	7.29	132.0810	2.31	↓^**^	↑^*^
20	Sphingolipids	SM [d18:1/22:1 (13Z)]	C_45_H_89_N_2_O_6_P	Neg	M + FA − H	27.13	829.6445	3.61	↑^*^	↓^*^
21	Sphingolipids	SM (d14:1/20:0)	C_39_H_79_N_2_O_6_P	Pos	M + Na	24.93	725.5566	1.84	↑^*^	↓^*^
22	Bile acid	Chenodeoxycholic acid	C_24_H_40_O_4_	Pos	M + NH_4_	13.84	410.3266	1.52	↑^*^	—
23	Fatty acid	Docosadienoate (22:2n6)	C_22_H_40_O_2_	Pos	M + H	17.64	319.2997	1.61	↑^**^	↑^**^
24	Other	1-Methyladenosine	C_11_H_15_N_5_O_4_	Pos	M + H	1.07	282.1197	2.63	↑^*^	↓^*^
25	Other	Adenosine 3′-monophosphate	C_10_H_14_N_5_O_7_P	Pos	M + H	1.10	348.0710	2.12	↑^**^	↓^**^
26	Other	Creatinine	C_4_H_7_N_3_O	Pos	M + H	1.00	114.0666	1.65	—	↓^**^

^*^p < 0.05, ^**^p < 0.01, and ^***^p < 0.001. CON, the control group; MOD, the model group; TAU, the taurine group.

**Table 3:** Trend of significantly differential metabolites in feces.

No.	Sort	Metabolite	Formula	Mode	Adducts	RT	m/z	VIP	Trend	
									MOD/CON	TAU/MOD
1	Amino acids	L-hisidine	C_6_H_9_N_3_O_2_	Pos	M + H	1.00	156.0771	1.87	↓^***^	↑^*^
2	Amino acids	L-phenylalanine	C_9_H_11_NO_2_	Neg	M − H	2.62	164.0717	2.15	↓^**^	↑
3	Amino acids	L-lysine	C_6_H_14_N_2_O_2_	Pos	M + H	0.93	147.1130	1.86	↓^**^	—
4	Amino acids	D-pipecolic acid	C_6_H_11_NO_2_	Pos	M + H	1.10	130.0866	1.72	↓^***^	↑^*^
5	Cholic acid	Alpha-muricholic acid	C_24_H_40_O_5_	Neg	M − H	10.28	453.2860	2.37	↑^***^	—
6	Cholic acid	Tauro-alpha-muricholic acid	C_26_H_45_NO_7_S	Pos	M + Na	6.38	480.2784	1.8	↑^**^	↑^*^
7	Cholic acid	Cholic acid	C_24_H_40_O_5_	Neg	M − H_2_O − H	13.09	389.2701	1.88	↑^**^	↑^*^
8	Cholic acid	Chenodeoxycholic acid	C_24_H_40_O_4_	Pos	M + NH_4_	13.84	410.3266	1.96	↑^**^	—
9	Cholic acid	Tauroursodeoxycholic acid	C_26_H_45_NO_6_S	Pos	M + H	10.78	464.2836	3.01	↑^*^	↑^**^
10	Cholic acid	Taurocholic acid 3-sulfate	C_26_H_45_NO_10_S_2_	Neg	M − H	5.48	296.6171	1.61	↑^*^	↑^***^
11	Cholic acid	Taurochenodeoxycholic acid	C_26_H_45_NO_6_S	Pos	M + H − H_2_O	10.78	482.2939	3.43	—	↑^**^
12	Cholic acid	Choline glycerophosphate	C_8_H_20_NO_6_P	Pos	M + H	1.00.	258.1105	2.7	↑^*^	↑^*^
13	Fatty acid	Eicosapentaenoic acid	C_20_H_30_O_2_	Neg	M − H	17.99	301.2173	2.1	↓^***^	↑
14	Fatty acid	Docosahexaenoic acid	C_22_H_32_O_2_	Neg	M − H	18.76	327.2330	2.05	↓^***^	↑
15	Fatty acid	2-hydroxyhexadecanoic acid	C_16_H_32_O_3_	Neg	M − H	20.36	271.2280	1.73	↑^***^	↑
16	Fatty acid	13 (S)-HODE	C_18_H_32_O_3_	Pos	M + H − H_2_O	14.92	279.2319	1.61	↓^***^	↓
17	Fatty acid	Tetracosahexaenoic acid	C_24_H_36_O_2_	Pos	M + H − H_2_O	14.99	339.2685	1.86	↑^**^	—
18	Fatty acid	Arachidonic acid	C_20_H_32_O_2_	Neg	M − H	18.86	303.2329	1.64	↓^**^	↑
19	Other	Raffinose	C_18_H_32_O_16_	Neg	M + FA − H	1.06	549.1674	2.92	↓^**^	↑^**^
20	Other	Acetylpterosin C	C_16_H_20_O_4_	Pos	M + H	5.99	277.1438	2.31	↑^***^	↑^*^
21	Other	Tetrahydrothiophenecarboxylic acid (THTC)	C_5_H_8_O_2_S	Pos	M + H	1.27	150.0586	1.62	↓^*^	↑^*^

^*^p < 0.05, ^**^p < 0.01, and ^***^p < 0.001. 13 (S)-HODE, (13S)-Hydroxyoctadecadienoic acid; CON, the control group; MOD, the model group; TAU, the taurine group.

**Table 4:** Trend of significantly differential metabolites in urine.

No.	Sort	Metabolite	Formula	Mode	Adducts	RT	m/z	VIP	Trend	
									MOD/CON	TAU/MOD
1	Amino acids	Indolelactic acid	C_11_H_11_NO_3_	Pos	M + H	3.92	206.0815	3.2	↓	↑^*^
2	Amino acids	1-Methylhistidine	C_7_H_11_N_3_O_2_	Pos	M + H	0.97	170.0928	1.71	—	↑^*^
3	Organosulfonic acids and derivatives	Taurine	C_2_H_7_NO_3_S	Neg	M − H	0.99	124.0073	1.77	↓^***^	↑^***^
4	Sphingolipids	Ceramide (d18:1/22:0)	C_40_H_79_NO_3_	Neg	M − H	26.89	666.6040	2.26	↓^*^	↓^*^
5	Other	Creatinine	C_4_H_7_N_3_O	Pos	M + H	1.00	114.0666	1.52	↑^***^	—
6	Other	2,5-octadien-1-ol	C_8_H_14_O	Pos	M + H	4.58	127.1121	2.15	↑^***^	↓^*^

^*^p < 0.05, ^**^p < 0.01, and ^***^p < 0.001. CON, the control group; MOD, the model group; TAU, the taurine group.

#### 3.2.3 KEGG pathway enrichment analysis identifies metabolic pathways affected by HFD and taurine

**Figure 3:**
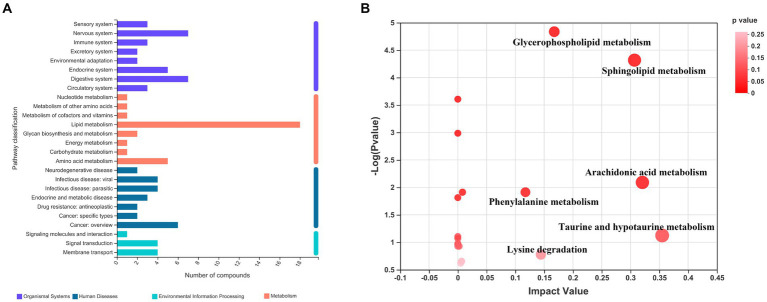
KEGG enrichment analysis and pathway analysis of the three groups of differential metabolites. **(A)** Pathway classification analysis by KEGG; **(B)** Pathway topology analysis.

In order to visualize the affected metabolic pathways, the differential metabolites of three groups were analyzed by KEGG (see text foot note 4). Pathway classification analysis (Figure 3A) revealed that 18 metabolites were classified under lipid metabolism, 5 under amino acid metabolism, 7 under nervous system function, 7 under digestive system function, 5 under the endocrine system, and 6 under cancer overview. We performed a topological analysis of metabolic pathways via MetaboAnalyst 5.0 (see text foot note 5) to explore which were most affected. As shown in Figure 3B, the larger the bubble, the more important the pathway is. Hyperlipidemia and taurine treatment had the greatest impact on 6 metabolic pathways (impact value >0.1), including taurine and hypotaurine metabolism, arachidonic acid metabolism, sphingolipid metabolism, glycerophospholipid metabolism, phenylalanine metabolism, and lysine degradation. The binding of bile acids to taurine is one of the main ways of taurine metabolism in the body. Although bile acid metabolism is not among the most impacted ones, we still include it in the discussion of taurine and hypotaurine metabolism.

## 4 Discussion

Previous studies have demonstrated that taurine supplementation has a preventive effect on hyperlipidemia and related diseases caused by a high-fat diet (7, 8). However, there are relatively few studies on the effects of taurine on hyperlipidemic metabolites and the possible underlying mechanisms. The present study has evaluated the effect of long-term supplementation of taurine on lipid metabolism disorder in HFD-fed mice by using the UPLC-MS untargeted metabolomics approach. Taurine significantly prevented the weight gain and lipid abnormalities (including increased TC, TG, LDL-C, and decreased HDL-C) induced by a high-fat diet in mice. It also alleviated the morphological damage and lipid deposition in the liver and adipose tissues of hyperlipidemic mice. These findings are consistent with those of other previously reported studies (13, 26). Based on the UPLC-MS untargeted metabolomics approach, we investigated the effects of taurine on metabolic profiling of serum, liver, urine, and feces in hyperlipidemia mice. A total of 76 potential metabolic markers were identified from the untargeted metabolomics data, including 23 in serum, 26 in liver, 6 in urine, and 21 in feces. The KEGG database and MetaboAnalyst pathway analysis indicated that these potential metabolic markers were mainly involved in bile acid and taurine metabolism, lipid metabolism and amino acids metabolism.

Cholesterol biotransformation to bile acids (BA) is critical for eventual removal of excess cholesterol and maintenance of cholesterol homeostasis (27). The primary BA, including cholic acid (CA) and chenodeoxycholic acid (CDCA), is produced from cholesterol in hepatocytes. Then taurine and glycine are conjugated with BA and are exported to the intestine. Taurocholic acid, a conjugate of taurine and BA, is the main metabolic form of taurine. Secondary BA, including lithocholic acid (LCA), deoxycholic acid (DCA) and their respective isoforms, are formed from the primary BA by bacteria in the colon. The BAs in the intestine contribute to the digestion and absorption of fat. In addition to their participation in the enterohepatic circulation, any excess bile acids present in the intestine are eliminated through fecal excretion. In our study, the fecal levels of CA, CDCA, alpha-muricholic acid, tauroursodeoxycholic acid, taurocholic acid 3-sulfate and tauro-alpha-muricholic acid in the MOD group increased (Table 3), suggesting increased synthesis and intestinal release of BA in response to HFD intake. In the taurine administration group, not only CA but also all kinds of taurine-conjugated BA (TAU-BA) further increased (Table 3). Numerous previous studies only showed that total BA production was augmented by taurine as one of the mechanisms for lowering cholesterol (18, 28), but here, we further revealed that taurine promoted the production of CA and all TAU-BA in BA. Alterations in BA profiles, particularly the increase of unconjugated and secondary BA, are associated with the gut microbiota after high-fat consumption, which may have potential unfavorable impacts on colonic and host cardiometabolic health in humans (29). The gut microbiota can directly alter bile acid composition through deconjugation of taurine by bile salt hydrolase (30). Here, fecal TAU-BAs increased significantly due to taurine administration, suggesting taurine may improve gut microbiota abnormalities under HFD conditions.

In addition, our research found that HFD induced the increase of hepatocytic CDCA and serum CDCA sulfate and 3α,7α-Dihydroxycoprostanic acid (as a precursor of CDCA). Nevertheless, taurine administration was found to effectively prevent the increase in serum CDCA sulfate and 3α,7α-Dihydroxycoprostanic acid (Tables 1, 2). In the cellular microenvironment, CDCA, CA, DCA, and LCA at physiological concentrations are known to activate farnesoid X receptor (FXR) which inhibits the transcription of CYP7A1 by negative feedback. The CDCA is the most potent ligand for this receptor (31). The reduction in serum CDCA sulfate and CDCA precursor, and the increase in fecal taurine-conjugated CDCA excretion, suggested a decrease in overall CDCA levels which may relieve the inhibition of CYP7A1 transcription.

Metabolite analysis showed that TG (12:0/16:1/16:0) and TG (12:0/14:0/16:1) were abnormally increased in the MOD group (Table 1). Taurine administration prevented the above two metabolites abnormalities, which was consistent with the serum biochemical test results. In most mammalian cells, phosphatidic acid can be dephosphorylated by phosphatidate phosphohydrolase (PAP) to form diglycerides (DGs), which serve as precursor molecules for the synthesis of TG, as well as phosphatidylcholines (PC) and phosphatidylethanolamines (PE) (32). Moreover, DGs also originated from the failure of esterification or breakdown of the triglycerides (TGs) (32). Compared with the CON group, the MOD group exhibited a significant increase in liver DGs and a significant decrease in serum DGs. However, TAU mice showed a significant decrease in liver DGs and a partial increase in serum DGs compared with the MOD group (Tables 1, 2). Masuda et al. (33) reported that high fat and high sugar diet increased DGs *de novo* production and accelerated DGs and TGs accumulation in the liver of mice. Excess DGs, the most important lipotoxins, induced chronic inflammation and damaged multiple organs and systems by entering alternative non-oxidative pathways (34). Moreover, it has been clear that high levels of intracellular DGs are correlated with steatosis and insulin resistance (33, 35, 36). As a basic membrane component and metabolic intermediate, DGs are involved in the regulation of many protein activities (37). In a high-fat microenvironment, abnormal intracellular DG increases promoted protein kinase C (PKC) activation (38) and decreased phosphatidylin-ositol-3-kinase (PI3K) activation (34), ultimately leading to insulin resistance. It is well known that PKC/MAPK/PI3K pathway is one of the vital signaling pathways in hyperlipidemia and its secondary diseases such as atherosclerosis or steatosis. Here we found that abnormally high levels of TGs and DGs in the liver were prevented with taurine administration. Das et al. (39) have revealed that taurine can repress the activation of PKC and mitogen-activated protein kinase (MAPK) in the diabetic kidney and alleviate the disease symptoms of diabetes. In our previous study, it is found that taurine increased phosphorylation of PI3K and protein kinase B (AKT) in steatotic HepG2 cell (40). All evidence suggests that taurine can alleviate intracellular DGs accumulation, thereby reducing tissue lipid deposition, and may regulate the relevant signaling pathways driven by DGs to prevent HFD-induced disease states.

Glycerophospholipids (GPs) are the most abundant lipid species in most mammalian cell membranes, which constitute cytoskeletal organization, maintain membrane fluidity and ion permeability, and play a crucial role in cell growth and stability (41). The major classes of GPs are PC, PE, phosphatidylinositols (PI), phosphatidylserines (PS) and cardiolipins (CL) (42). Mammalian cells maintain the homeostasis of GPs within narrow limits, which means that deviations from optimal composition are detrimental (43). Our results indicated that, compared with the CON group, except PE (P-16:0/22:6), other GPs such as PE (16:0/0:0), PS [16:0/18:2 (9Z, 12Z)], PI [16:0/22:5 (7Z, 10Z, 13Z, 16Z, 19Z)], PC (14:0/0:0), GPCho (16:1/16:1) and CL (a-13:0/a-25:0/18:2/18:2) were all increased in the group (Tables 1, 2), which was consistent with previous studies (44, 45). In addition, the metabolites of GPs in MOD group were also disturbed, LysoPC (20:0/0:0) decreased (Table 1) and LysoPE [16:1 (9Z)/0:0] increased (Table 2). However, in TAU group, the abnormal changes of above metabolites were significantly prevented. Many studies have shown that GPs and their metabolites were significantly associated with obesity, T2D, atherosclerosis, and other metabolic diseases (46–48). The GPs and their subclasses are even considered potential differential metabolites for aging-related diseases, oxidative stress and chronic inflammation (48, 49). For example, large amounts of PC delivered to the liver from circulating HDL-C (~25%) and LDL-C (~50%) are converted to TG in mouse hepatocytes, possibly contributing to the development of steatosis (50, 51). PE is an important cofactor and membrane component, and PE with arachidonic acid acyl chains is the target of lipoxygenase, whose oxidation causes lipotoxicity and promotes ferroptosis (52). Thus, it is reasonable to speculate that taurine may block the development of liver damage through preventing the abnormal increase of GPs caused by HFD. Furthermore, in a recent study, HFD promoted the activation of aryl hydrocarbon receptor (AhR) by exosome-derived PCs, resulting in the repressed expression of insulin receptor substrate 2 (IRS2) and its downstream genes PI3K and AKT, leading to insulin resistance (53). Li et al. (54) reported that taurine prevented the reduction of AKT and phosphorylated AKT levels in hyperglycemic kidneys. Another study has also found that taurine can block the decline in the phosphorylation of AKT and PI3K in the liver of diabetes animals, thus facilitating the uptake of glucose (55). Furthermore, taurine up-regulated phosphorylation levels of PI3K, Akt, and mTOR in sciatic nerve of diabetic rats and dorsal root ganglion neurons exposed to high glucose (56). It is again suggested that taurine can prevent the abnormal GPs changes caused by HFD, potentially serving as both a mechanism for reducing tissue damage and a key regulator of signaling molecules associated with the anti-hyperlipidemic effect.

Sphingolipids (also known as sphingomyelins, SMs) is one of the most active lipids ubiquitously produced in eukaryotic cells. In this study, the levels of SM (d14:1/20:0), SM [d18:1/22:1 (13Z)] and glucosylceramide (d18:1/22:0) in the MOD group were remarkably higher than that of the CON group (Tables 1, 2). However, after taurine administration, increased levels of SM (d14:1/20:0) and SM [d18:1/22:1 (13Z)] were prevented, although the other above-mentioned SMs and their metabolites did not change significantly. Altered metabolism and ectopic accumulation of SMs in different cellular microenvironments have been shown to contribute to pathological conditions such as hyperlipidemia, diabetes, insulin resistance, and oxidative stress (55, 57). Many studies have shown that inhibition of *de novo* sphingolipid synthesis reduced TG accumulation in the liver and the development of insulin resistance (58–60). Recent studies of hyperlipidemia and T2D support the conclusion that increased intracellular sphingolipid content may impair mitochondrial function by interfering with respiratory chain activity and other aspects related to mitochondrial bioenergetics (57). There is evidence that taurine plays a role in maintaining mitochondrial function (61). Sphingolipid accumulation negatively affects phosphorylation of AKT leading to decreased glucose uptake in skeletal muscle as well as increased gluconeogenesis and glycogenolysis in liver (62). As described above, several studies have shown that taurine can up-regulated phosphorylation levels of PI3K and AKT in various tissues of diabetes animals. The effect of taurine on mitochondrial function and the activity of signal molecules such as AKT seem to be related to the level of SMs in tissues. Therefore, taurine can partially prevent sphingolipid metabolic disorder caused by HFD, potentially thereby modulating relevant signaling pathways to improve tissue damage in hyperlipidemia.

Eicosapentaenoic acid (EPA), docosahexaenoic acid (DHA), and arachidonic acid (ARA) are three kinds of PUFA that are beneficial for CVD prevention (63). This study showed the levels of EPA, DHA and ARA in feces, as well as DHA and 12,13-epoxy-9-octadecenoic acid levels in serum, were significantly reduced in the MOD group (Tables 1, 3). Some intestinal microbes can produce PUFA, which can directly or indirectly affect intestinal microecology and maintain the basic physiological characteristics of the intestines (64). However, an HFD causes imbalance in intestinal flora and worsens the onset of lipid metabolism disorders (65). Taurine administration showed a preventive trend against the reduction of the above-mentioned PUFAs in serum induced by a HFD, but particularly leading to a significant increase in DHA. Taurine also inhibited the decrease of polyunsaturated fatty acids in feces, but this was not statistically significant. Increased EPA and DHA could significantly increase the HDL-C/apoA-I ratio and HDL-C levels, expand fatty acid degradation, and reduce the levels of plasma low-density lipoprotein cholesterol (VDL-C), LDL-C and TG, thus improving lipid profiles (66, 67). Clinical studies have also demonstrated that EPA and DHA dose-dependently reduce plasma TG levels and the risk of CVD (63, 68). Therefore, increasing serum DHA level may be one of mechanisms of taurine’s anti-hyperlipidemia effect.

Many studies have shown that hyperlipidemia is often accompanied by disorders of amino acid metabolism, especially the reduction of the levels of amino acids and their derivatives (69, 70). Our study showed that L-histidine, L-lysine and L-phenylalanine in feces decreased in the MOD group (Table 3). Furthermore, the levels of 2-methylindole (tryptophan derivative) in liver, 1-methylhistidine and indolelactic acid (tryptophan derivative) in urine reduced in the MOD group (Tables 2, 3). After administration of taurine, L-phenylalanine and L-histidine in feces, 2-methylindole and tiglylglycine in liver, 1-methylhistidine in urine and serum, and indolelactic acid in urine were significantly increased than that of MOD group (Tables 1–4). HFD-induced obesity leads to amino acid catabolism in the gut and impairs amino acid diversity (71). It has been reported that alterations in the gut microbiota in obese T2D subjects can affect the bioavailability of host amino acids, which may lead to metabolic disorders (72). In this study, the improving effect of taurine on certain amino acids (especially histidine, tryptophan, and its derivatives) levels was identified for the first time, which may be due to the improvement of intestinal microbial environment by taurine. Indolelactic acid is produced by gut microbes metabolizing tryptophan through tryptophanase (29). Studies have shown that indolelactic acid is closely related to the regulation of systemic immune cell function, which can inhibit the expression of pro-inflammatory cytokines and excessive inflammatory responses in macrophages (73). Obesity and inflammatory symptoms induced by HFD are frequently associated with chronic inflammatory lesions dominated by infiltrating macrophages (74). The turn of indole lactate levels suggests the inhibitory effect of taurine on inflammation. Moreover, histidine is an essential amino acid with many benefits for human health, while its concentrations decrease in pathological conditions, such as chronic kidney disease (75). Histidine and variation in creatinine levels reflected the protective effect of taurine on liver and kidney functions (40). In addition, metabolites drive pivotal covalent chemical modifications of DNA and RNA (such as methylation) and of proteins (post-translational modifications) (19). Here, the methylation of histidine and indole increased significantly by taurine, which may provide a favorable microenvironment for protein methylation. All the above results suggested that taurine can partially prevent HFD-induced abnormal variations of amino acids, which is beneficial to improve the tissue damage and inflammatory lesions associated with hyperlipidemia.

**Figure 4:**
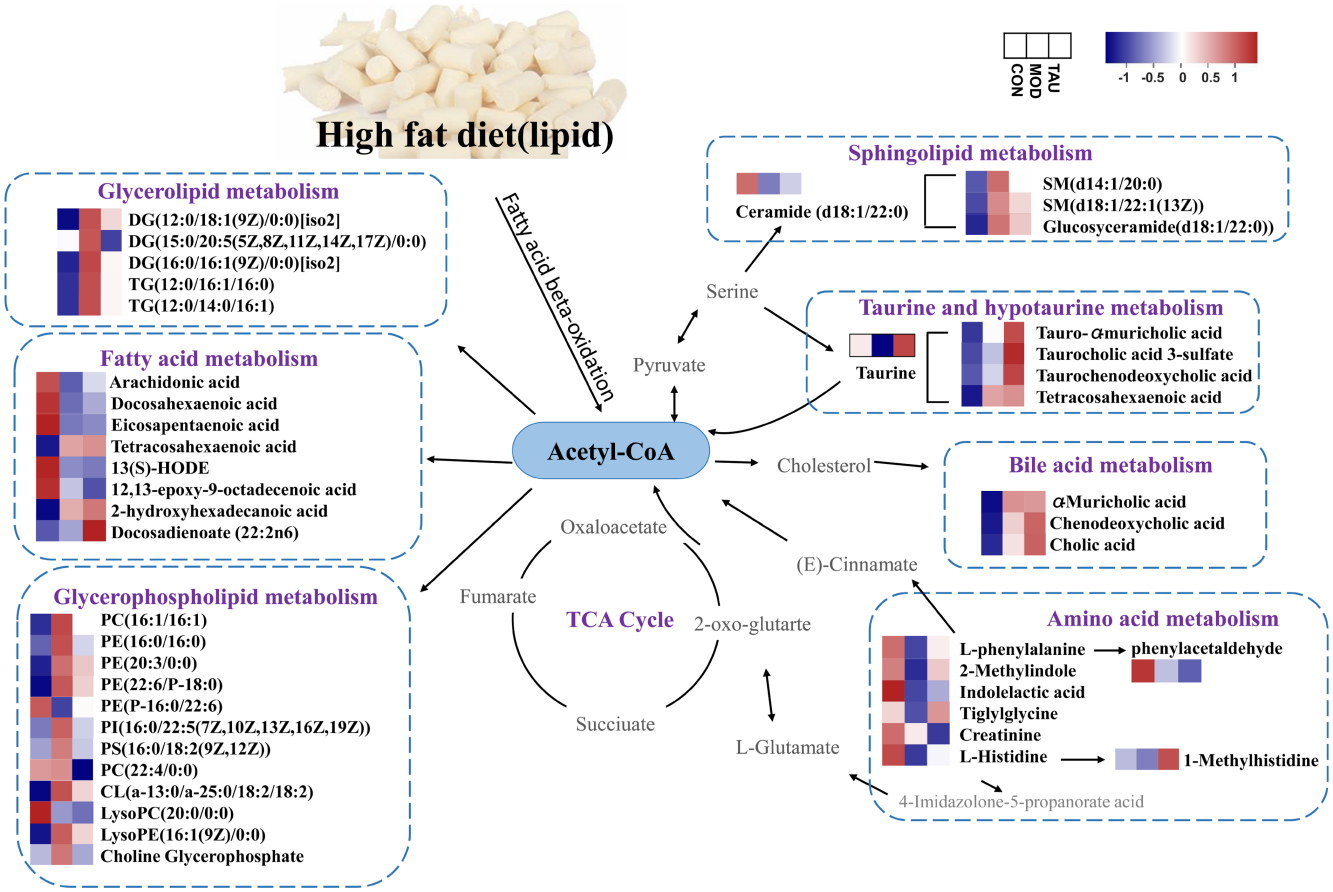
Schematic diagram of modulating biomarkers and potentially disturbed metabolic pathways.

The potential anti-hyperlipidemia mechanism of taurine was identified by comprehensively analyzing the metabolic pathway of differential metabolites in serum, liver, urine, and feces. Furthermore, differential metabolites can modify and interact with proteins and DNA depending on the activity of their respective enzymes, thereby altering the proteome and the epigenome (19). In hyperlipidemic conditions, a large number of fatty acids were converted into acetyl-CoA. Acetyl-CoA, as an indicator of the cell’s nutritional state, determines the balance between cellular catabolism and anabolism by simultaneously operating as a metabolic intermediate and as a second messenger (76). Under conditions of carbohydrate excess, Acetyl-CoA will be directed away from TCA cycle of the mitochondria and back to the cytosol for the synthesis of fatty acids and sterols, driving the storage of excess carbohydrates as fat (77). As shown in Figure 4, HFD increased the levels of nearly all identified lipid metabolites and decreased several healthful PUFAs, indicating HFD can elevate fat synthesis, such as GP, SM, DG and TG, but reduce the synthesis of PUFA. Taurine administration may mainly switch the anabolism to catabolism, inhibit the synthesis of above lipid metabolites and catabolize them into TCA cycle, and accelerate the biotransformation of cholesterol into BA and intestinal excretion as conjugated BA. Taurine can promote the synthesis of PUFAs yet. Moreover, taurine can prevent the disorder of amino acid metabolism caused by HFD, promote the synthesis of amino acids and their derivatives, and maintain amino acid homeostasis.

Taurine can prevent the abnormalities of metabolites such as DGs, GPs and SMs induced by high-fat diet, and regulate fatty acid metabolism, sphingolipid metabolism, glycerophospholipid metabolism, diacylglycerol metabolism, amino acid metabolism, bile acid and taurine metabolism, taurine and hypotaurine metabolism. Regulation of these metabolic pathways may play the role of active drivers in biological processes and regulate the expression and activity of vital signaling macromolecules involved in the pathological progress or tissue damage of hyperlipidemia. However, further studies are needed to confirm the levels of target genes or proteins associated with altered metabolic pathways and to demonstrate at the molecular level how taurine reduces blood lipids.

## 5 Conclusion

To date, we have a limited understanding of the effect of taurine on hyperlipidemic metabolites. Our study showed that taurine can partially prevent the disorder of metabolites in hyperlipidemia, mainly classifying as BAs, GPs, SMs, DGs, TGs and PUFAs. The treatment of taurine may affect fatty acid metabolism, sphingolipid metabolism, glycerophospholipid metabolism, diacylglycerol metabolism, amino acid metabolism, bile acid and taurine metabolism, taurine and hypotaurine metabolism. It indicates that taurine can activate metabolites including DGs, GPs, and SMs, which may facilitate various anti-hyperlipidemia signal pathways. The study provided new evidence for the possible molecular mechanisms and targets of taurine for anti-hyperlipidemia therapies. Although the direct evidence for the causality between metabolite and signaling pathways is insufficient, our results still suggest that efforts to elaborate the beneficial effect of taurine could be a promising way to prevent hyperlipidemia.

## Data availability statement

The raw data supporting the conclusions of this article will be made available by the authors, without undue reservation.

## Ethics statement

This research protocol was approved by the Experimental Animal Ethics Committee of the Functional Test Center for Health Food, College of Arts and Sciences, Beijing Union University (Approval Number: 20220301).

## Author contributions

XG: Writing – original draft, Investigation, Supervision, Writing – review & editing. TO: Methodology, Investigation, Writing – review & editing. XY: Validation, Supervision, Writing – review & editing. QS: Methodology, Writing – review & editing. LZ: Supervision, Writing – review & editing. SM: Conceptualization, Project administration, Writing – review & editing. JZ: Resources, Conceptualization, Project administration, Writing – review & editing. YZ: Conceptualization, Project administration, Writing – review & editing. WC: Conceptualization, Supervision, Writing – review & editing. JG: Conceptualization, Funding acquisition, Resources, Writing – review & editing, Supervision.

## Footnotes

^1^http://www.hmdb.ca/

^2^https://metlin.scripps.edu/

^3^https://docs.scipy.org/doc/scipy/

^4^https://www.kegg.jp/kegg/pathway.html

^5^https://www.metaboanalyst.ca/
