# Supplementary material for: Untargeted metabolomics based on ultra-high performance liquid chromatography-mass spectrometry/MS reveals the lipid-lowering mechanism of taurine in hyperlipidemia mice
Source: Front Nutr. 2024 Apr 19;11:1367589. doi: 10.3389/fnut.2024.1367589 (PMC11066166; doi:10.3389/fnut.2024.1367589)
Supplement: Supplementary file 1 [file Image_1.pdf]

Support Information

Support Information Figure S1: **Feed intake of mice**

Support Information Figure S2: **Typical UPLC-MS total ion chromatograms of serum samples.**

Support Information Figure S3: **Typical UPLC-MS overlapped total ion chromatograms of QC samples in positive and negative ion modes.**

Support Information Figure S4: **Typical UPLC-MS total ion chromatograms of liver samples.**

Support Information Figure S5: **Typical UPLC-MS total ion chromatograms of feces samples.**

Support Information Figure S6: **Typical UPLC-MS total ion chromatograms of urine samples.**

Support Information Figure S7: **Multivariate statistical analysis of UPLC-MC-based effects for taurine on liver from high-fat-fed mice and heatmaps of the identified metabolites.**

Support Information Figure S8: **Multivariate statistical analysis of UPLC-MC-based effects for taurine on urine from high-fat-fed mice and heatmaps of the identified metabolites.**

Support Information Figure S9: **Multivariate statistical analysis of UPLC-MC-based effects for taurine on feces from high-fat-fed mice and heatmaps of the identified metabolites.**

Abbreviations: TG, triglyceride; TC, total cholesterol; LDL-C, low-density lipoprotein cholesterol; HDL-C, high-density lipoprotein cholesterol; CVD, cardiovascular diseases; LC-MS, liquid chromatography coupled with mass spectrometry; UPLC-MS, ultra-high performance liquid chromatography-mass spectrometry; SPF, specific pathogen free; AI, atherosclerotic index; SREBP-1c, sterol regulatory element binding protein 1c; BA, bile acids; HFD, high-fat diet; PCA, Principal component analysis; OPLS-DA, orthogonal partial least squares discriminant analysis; SPE, solid phase extraction; EP, Eppendorf; ESI+, electrospray ionization in positive; ESI-, electrospray ionization in negative; RT, retention times; M/Z, mass charge ratio; VIP, variable importance projection; HMDB, Human Metabolome Database; SD, standard deviation; CON, control group; MOD, model group; TAU, high-dose taurine group; H&E, hematoxylin and eosin; TIC, total ion chromatograms; GPs, Glycerophospholipids; PC, phosphatidylcholines; PE, phosphatidylethanolamines; PI, phosphatidylinositols; PS, phosphatidylserines; CL, cardiolipins; CYP7A1, Cytochrome P450 Family 7 Subfamily A Member 1; SM, Sphingomyelin; LysoPC, lyso-phosphatidylcholine; LysoPE, lyso-phosphatidylethanolamine; DGs, diacylglycerols; GPCho, phosphatidylcholine; GPEtn, phosphatidylethanolamine; 13(S)-HODE, (13S)-Hydroxyoctadecadienoic acid; CA, cholic acid; AKT: protein kinase B； CDCA, chenodeoxycholic acid; LCA, lithocholic acid; DCA, deoxycholic acid; TAU-BA, taurine-conjugated bile acids; FXR, farnesoid X receptor; SMs, sphingolipids; PUFAs, polyunsaturated fatty acids; PKC, protein kinase C; PI3K, phosphatidylin-ositol-3-kinase; MAPK, mitogen-activated protein kinase; T2D, type 2 diabetes; AhR, aryl hydrocarbon receptor; IRS2, insulin receptor substrate 2; TCA, tricarboxylic acid； (PGC)-1α: Peroxisome proliferator-activated receptor-gamma coactivator, AMPK: adenosine monophosphate-activated protein kinase； LDLR: low density lipoprotein receptor；LXR: liver X receptor

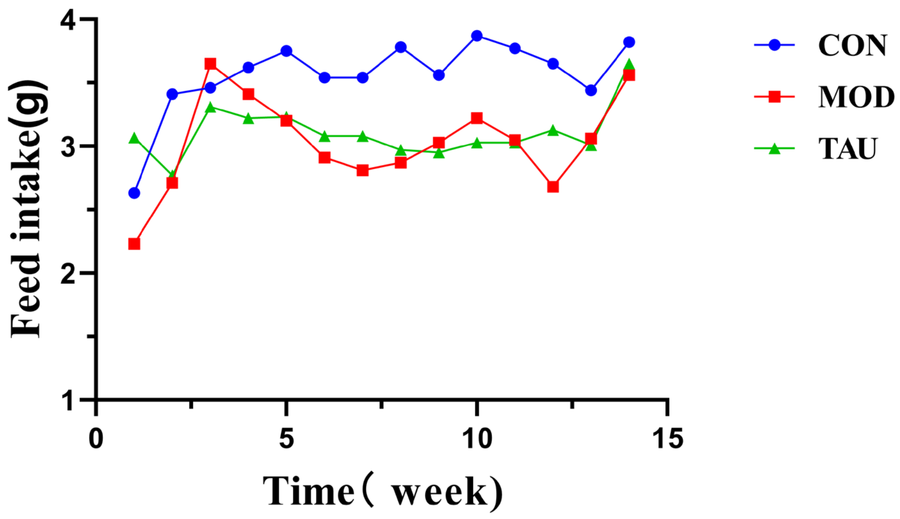

Support Information Figure S1: **Feed intake of mice**

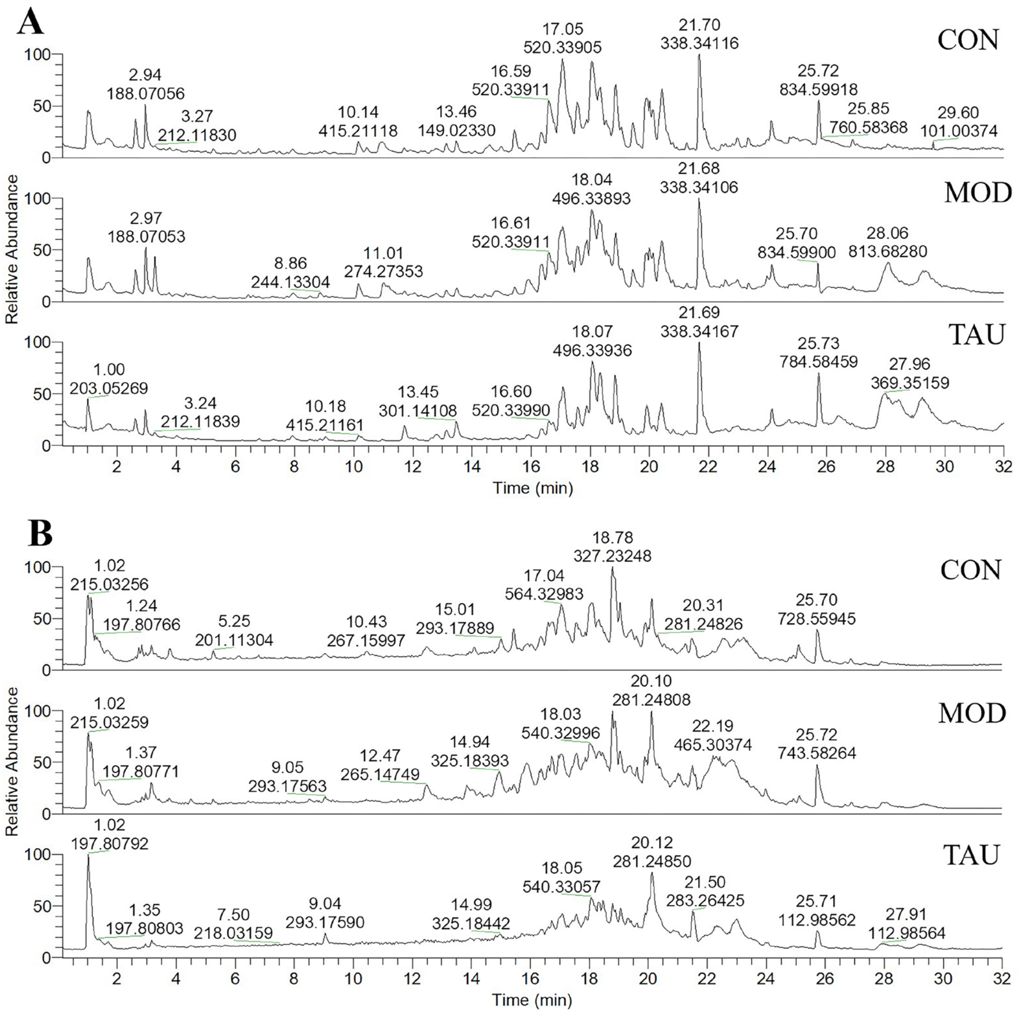

Support Information Figure S2: **Typical UPLC-MS total ion chromatograms of serum samples.** A) Positive ion mode; B) Negative ion mode.

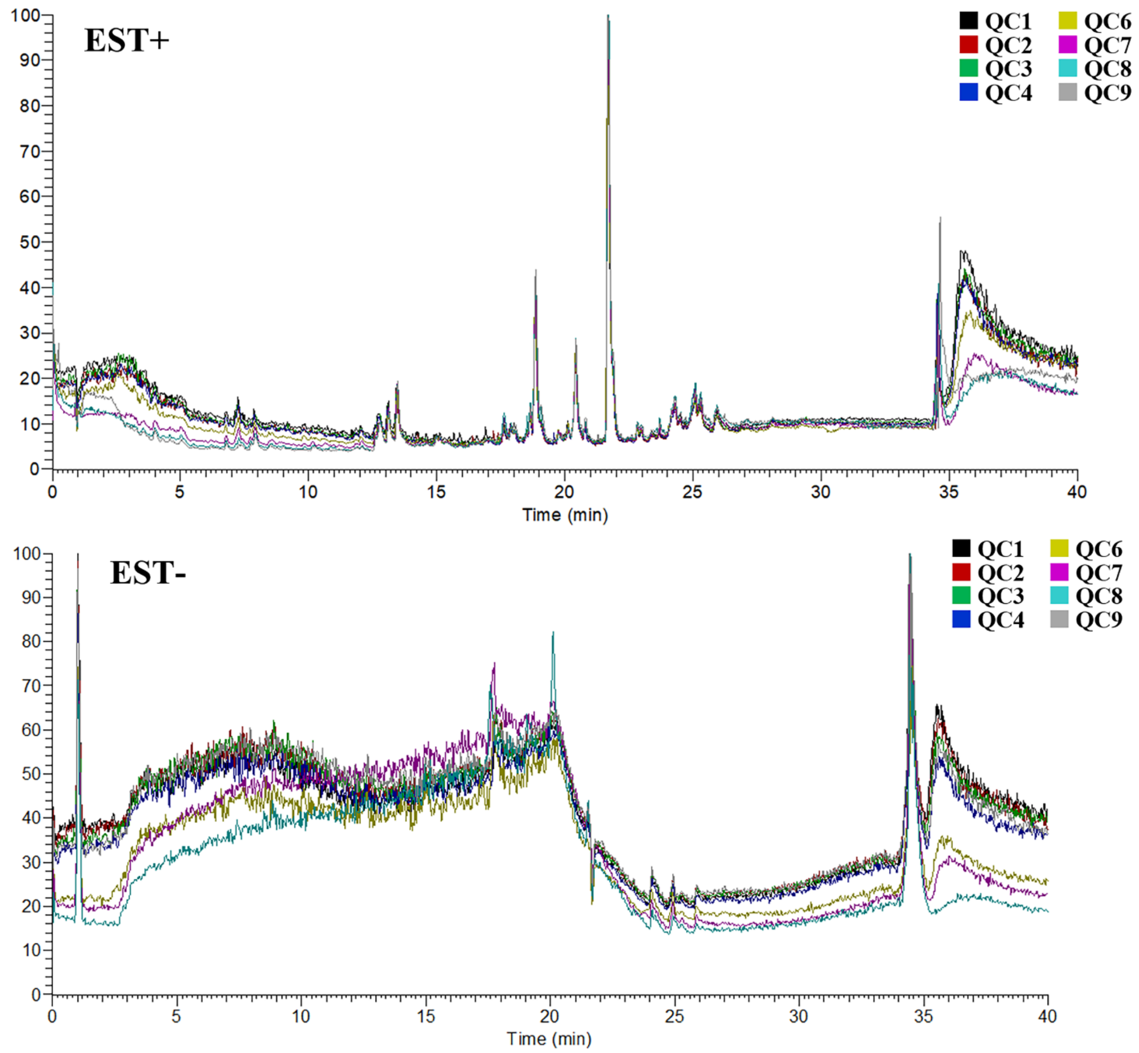

Support Information Figure S3: **Typical UPLC-MS overlapped total ion chromatograms of QC samples in positive and negative ion modes.**

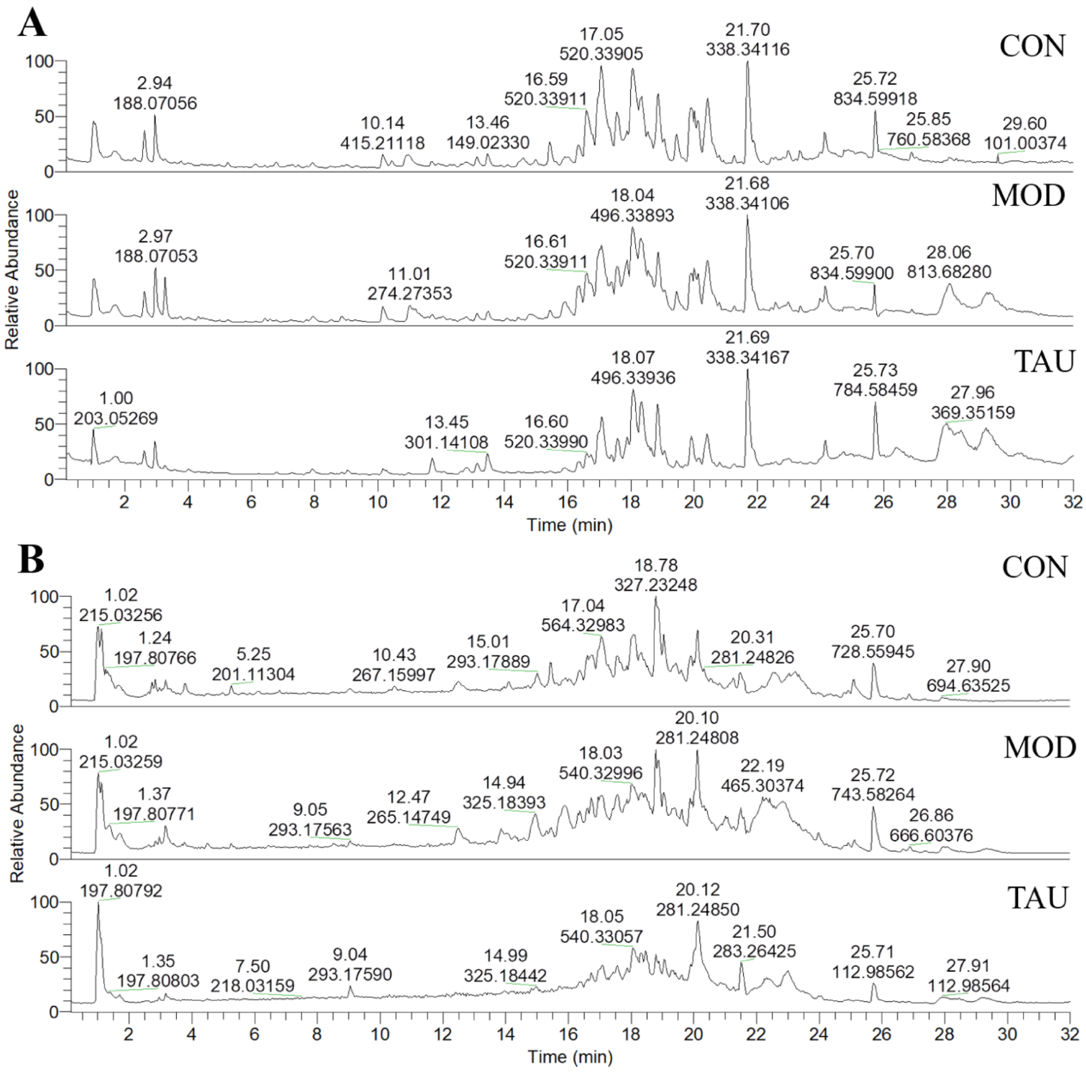

Support Information Figure S4: **Typical UPLC-MS total ion chromatograms of liver samples.**

A) Positive ion mode; B) Negative ion mode.

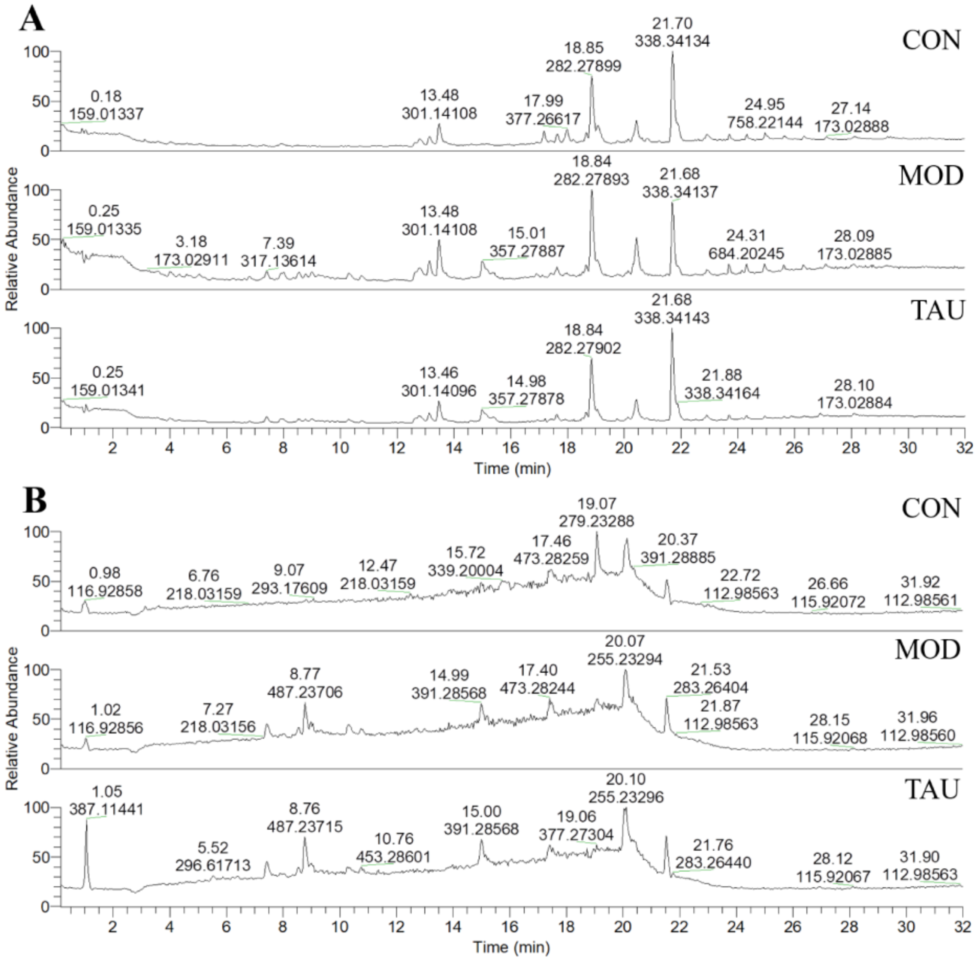

Support Information Figure S5: **Typical UPLC-MS total ion chromatograms of feces samples.**

A) Positive ion mode; B) Negative ion mode.

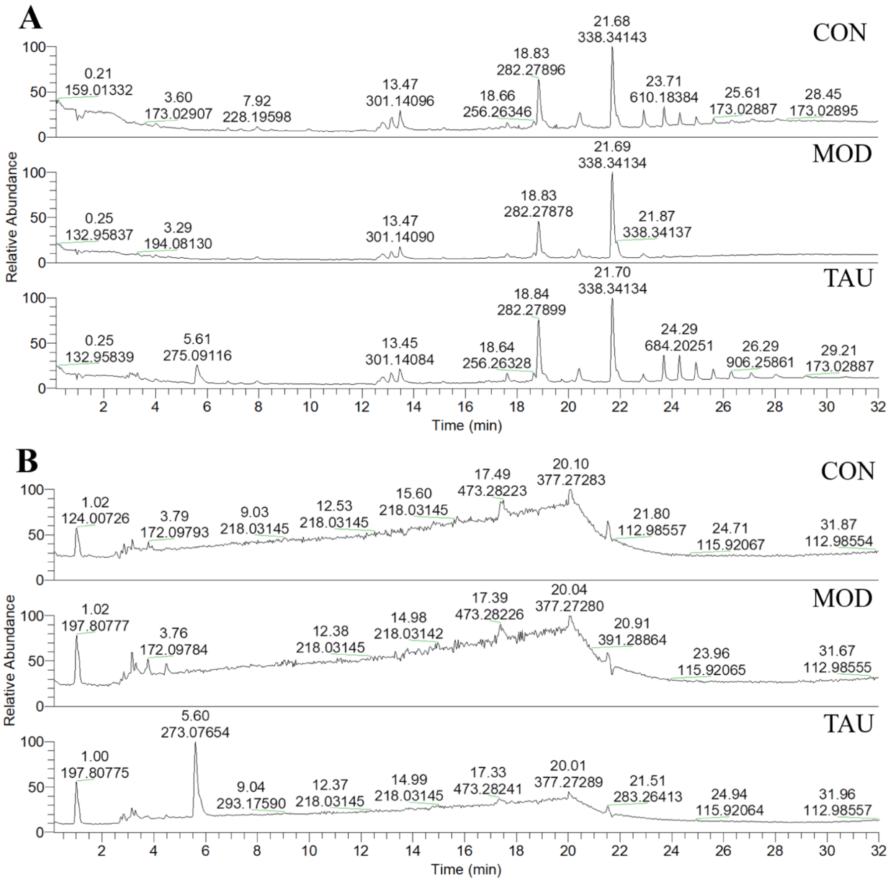

Support Information Figure S6: **Typical UPLC-MS total ion chromatograms of urine samples.** A) Positive ion mode; B) Negative ion mode.

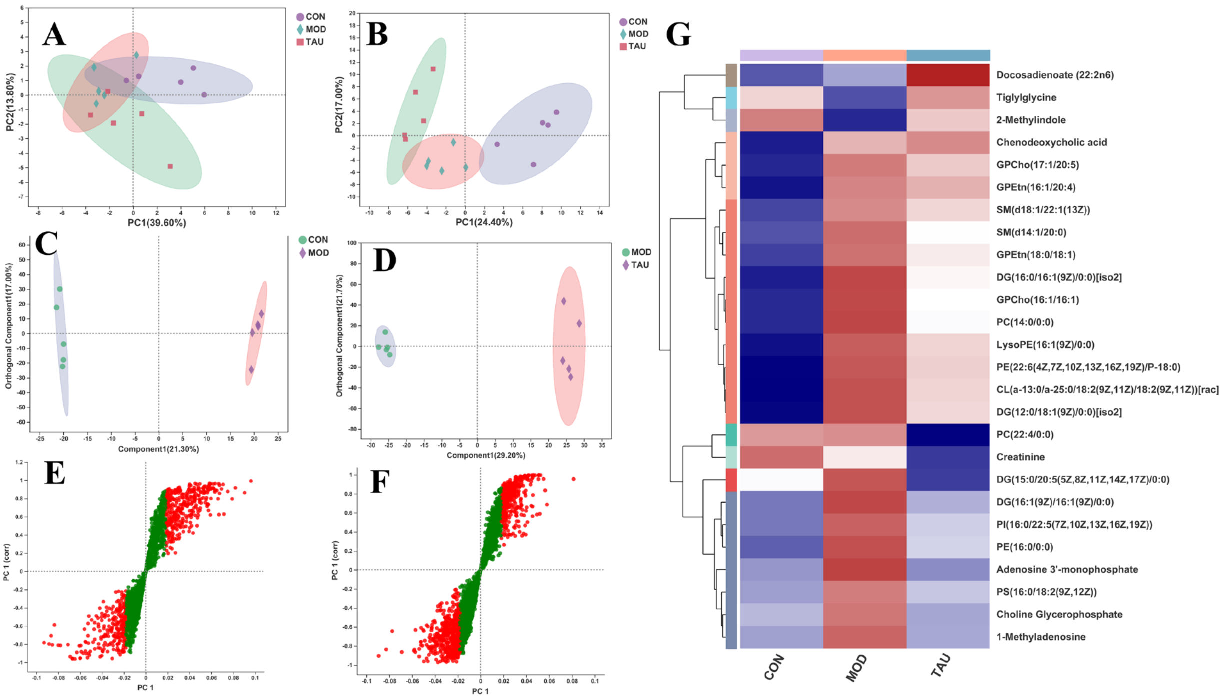

Support Information Figure S7: **Multivariate statistical analysis of UPLC-MC-based effects for taurine on liver from high-fat-fed mice and heatmaps of the identified metabolites.** A, B) Scatter plot of PCA scores in positive (A) and negative (B) ion mode; C) OPLS-DA score scatter plot comparing CON and MOD; D) OPLS-DA score scatter plot comparing TAU and MOD; E) S-plots comparing CON and MOD; F) S-plots comparing TAU and MOD. G) Hierarchical clustering heat map of the differential metabolites between CON, MOD, and TAU groups.

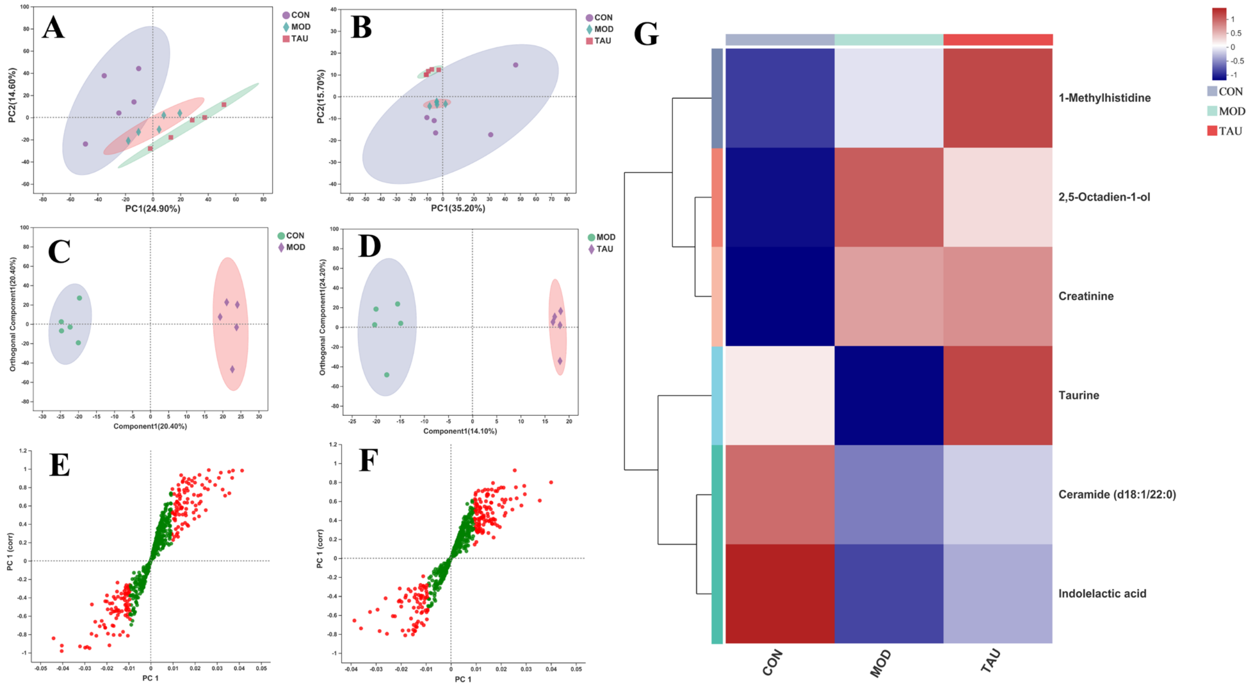

Support Information Figure S8: **Multivariate statistical analysis of UPLC-MC-based effects for taurine on urine from high-fat-fed mice and heatmaps of the identified metabolites.** A, B) Scatter plot of PCA scores in positive (A) and negative (B) ion mode; C) OPLS-DA score scatter plot comparing CON and MOD; D) OPLS-DA score scatter plot comparing TAU and MOD; E) S-plots comparing CON and MOD; F) S-plots comparing TAU and MOD. G) Hierarchical clustering heat map of the differential metabolites between CON, MOD, and TAU groups.

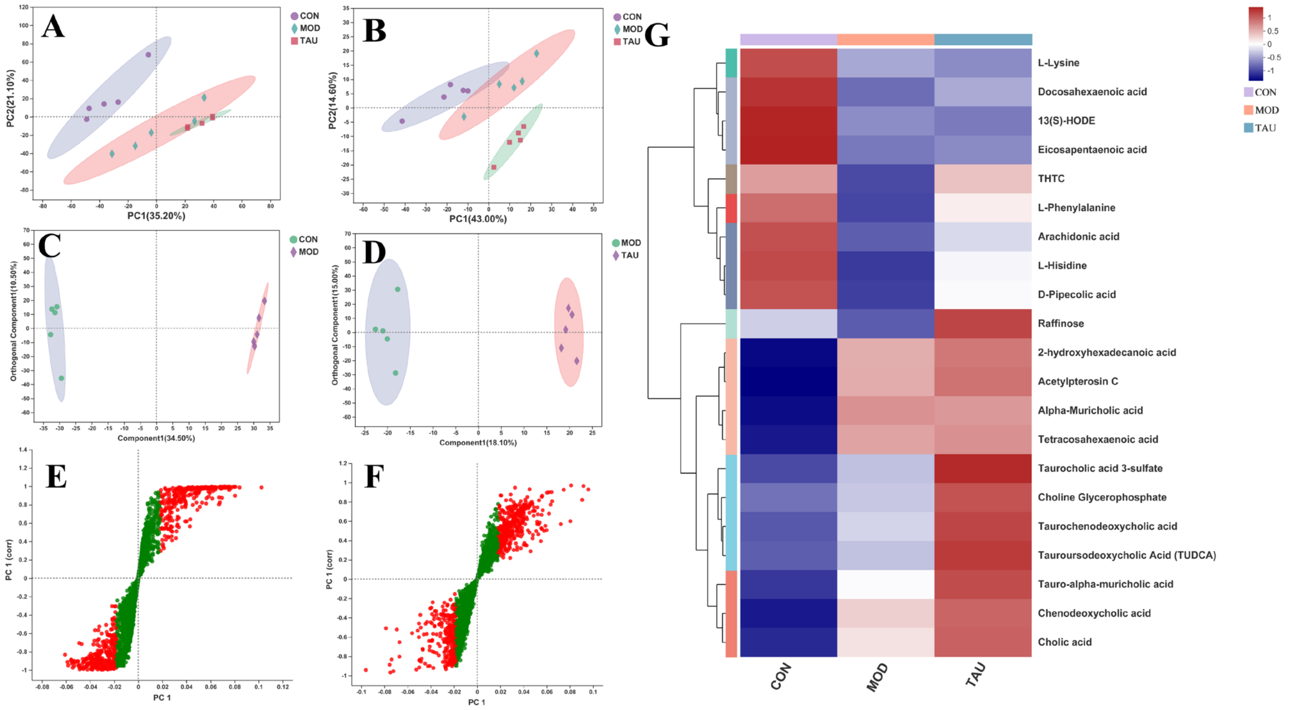

Support Information Figure S9: **Multivariate statistical analysis of UPLC-MC-based effects for taurine on feces from high-fat-fed mice and heatmaps of the identified metabolites.** A, B) Scatter plot of PCA scores in positive (A) and negative (B) ion mode; C) OPLS-DA score scatter plot comparing CON and MOD; D) OPLS-DA score scatter plot comparing TAU and MOD; E) S-plots comparing CON and MOD; F) S-plots comparing TAU and MOD. G) Hierarchical clustering heat map of the differential metabolites between CON, MOD, and TAU groups.
